# Immune Checkpoint Inhibitor‐Induced Diabetic Ketoacidosis in a Patient With Pre‐Existing Type 2 Diabetes: A Case Report

**DOI:** 10.1002/ccr3.73834

**Published:** 2026-11-03

**Authors:** Lingfen Zeng, Jieming Sun

**Affiliations:** ^1^ Department of Internal Medicine The Third People's Hospital of Baiyun District Guangzhou Guangdong China; ^2^ Department of Clinical Laboratory The Third People's Hospital of Baiyun District Guangzhou Guangdong China

**Keywords:** diabetic ketoacidosis, immune checkpoint inhibitor, immune‐related adverse event, PD‐1 inhibitor, toripalimab, type 2 diabetes

## Abstract

Immune checkpoint inhibitors (ICIs) may cause rare life‐threatening ICI‐associated diabetes mellitus. We report a 60‐year‐old Chinese male with pre‐existing type 2 diabetes who developed toripalimab‐induced diabetic ketoacidosis after 3 treatment cycles. He also had toripalimab‐related hyperthyroidism. Clinicians should closely monitor plasma glucose during and after ICI therapy.

## Key Clinical Message

Patients with pre‐existing type 2 diabetes receiving PD‐1 inhibitors are at risk of abrupt beta‐cell failure and diabetic ketoacidosis. Clinicians should monitor glucose throughout immunotherapy and after discontinuation, and the presence of one endocrine immune‐related adverse event should raise suspicion for others.

## 1 Introduction

Immune checkpoint inhibitors (ICIs), including PD‐1/PD‐L1 inhibitors and CTLA‐4 inhibitors, have revolutionized the treatment of advanced malignancies. However, they are associated with a spectrum of immune‐related adverse events (irAEs). ICI‐associated diabetes mellitus (ICI‐DM) is a rare but potentially life‐threatening irAE, with an estimated incidence of approximately 0.45% [1]. It is characterized by rapid‐onset insulin deficiency and often presents with diabetic ketoacidosis (DKA). Most affected patients require long‐term or lifelong insulin replacement therapy [2]. This complication is more commonly observed in patients receiving PD‐1/PD‐L1 pathway blockade, and its pathogenesis is closely linked to the disruption of PD‐1/PD‐L1‐mediated immune tolerance, leading to cytotoxic T‐cell infiltration and destruction of pancreatic beta‐cells [3, 4].

Herein, we report a case of DKA induced by toripalimab (a PD‐1 inhibitor) in a patient with pre‐existing type 2 diabetes. The patient developed DKA after only three cycles of toripalimab, with near‐complete beta‐cell failure as evidenced by an undetectable C‐peptide level. This case underscores the importance of vigilant glucose monitoring in patients receiving ICI therapy, especially those with pre‐existing diabetes.

## 2 Case History

A 60‐year‐old Chinese male (height 170 cm, weight 52 kg) was admitted to our hospital with a three‐day history of dry mouth, fatigue, increased urine output, palpitations, and poor appetite. He had no chest tightness, chest pain, abdominal pain, nausea, or vomiting. His bowel movements were normal, and his general mental status was fair.

The patient had a one‐year history of type 2 diabetes, with a maximum recorded blood glucose of 12.3 mmol/L. He had no classic symptoms of polyuria, polydipsia, polyphagia, or weight loss at the time of initial diagnosis. He was previously diagnosed with type 2 diabetes at another hospital and was maintained on metformin 0.5 g three times daily and acarbose 100 mg three times daily, and his glycemia had been well controlled.

His past medical history was significant for bladder cancer diagnosed six months prior to admission. He received toripalimab (240 mg intravenously every three weeks) for 21 days before undergoing cystectomy, and continued toripalimab postoperatively. One month after initiating toripalimab, he developed hyperthyroidism and took methimazole for one month at another hospital, which was then discontinued.

## 3 Investigations and Treatment

On admission, his vital signs were stable. Laboratory tests revealed: blood glucose 31.1 mmol/L, pH 7.11, PaCO_2_ 17.4 mmHg, total CO_2_ 6.1 mmol/L, base excess −21.3 mmol/L, standard bicarbonate 9.6 mmol/L, and actual bicarbonate 5.6 mmol/L. Fasting C‐peptide was undetectable at < 0.06 nmol/L. Serum beta‐hydroxybutyrate was markedly elevated at 8566 umol/L, confirming significant ketonemia and supporting the diagnosis of diabetic ketoacidosis. Routine blood counts and biochemistry were otherwise unremarkable, with glycated hemoglobin (HbA1c) of 8.2%. Diabetes‐associated autoantibodies (including anti‐GAD, IA‐2, and ICA) were negative. Abdominal ultrasound showed a normal pancreas.

The differential diagnosis for this presentation included hyperosmolar hyperglycemic state (HHS), sepsis‐associated metabolic acidosis, and lactic acidosis. However, the presence of significant ketonemia (inferred from the high anion gap metabolic acidosis and severe insulin deficiency), an undetectable C‐peptide level, and the absence of infectious or hypoperfusion evidence favored diabetic ketoacidosis over these alternatives.

He was promptly treated with intravenous fluid resuscitation, continuous insulin infusion, and supportive care. Human regular insulin was administered via continuous intravenous infusion at an initial rate of 5 U/h. The total fluid replacement volume within the first 24 h of admission was 5750 mL, and 3 g of potassium chloride was supplemented intravenously over the same period. His blood glucose gradually stabilized, serum ketones became negative, and repeat arterial blood gas analysis normalized. After resolution of ketoacidosis, the patient was transitioned to subcutaneous insulin therapy with insulin glargine 18 U once daily at bedtime combined with insulin aspart 8 U three times daily before meals, which achieved satisfactory glycemic control with plasma glucose fluctuations between 4.0 and 8.5 mmol/L.

## 4 Outcome and Follow‐up

At discharge, due to the patient's preference to avoid four daily insulin injections, he was transitioned from the basal‐bolus regimen to three times daily premixed insulin aspart 30, administered at 16 U before breakfast, 10 U before lunch, and 16 U before dinner subcutaneously. Glycemic control on this regimen was moderate, with plasma glucose fluctuating between 6 and 12 mmol/L. At the most recent follow‐up on October 13, with poorly controlled blood glucose fluctuating from 3.8 to 20.2 mmol/L and HbA1c of 8.8%, both fasting and 2‐h postprandial C‐peptide levels remained undetectable at < 0.06 nmol/L, indicating persistent near‐complete beta‐cell failure. Given suboptimal glycemic control on the premixed regimen, the patient is planned to be switched back to a basal‐bolus insulin regimen for improved glycemic management. Given the near‐complete and sustained beta‐cell failure evidenced by persistently undetectable C‐peptide, lifelong insulin therapy is anticipated. The patient was advised to continue regular glucose monitoring and follow‐up in the endocrinology clinic.

## 5 Discussion

Our patient developed DKA after three cycles of toripalimab, despite having pre‐existing type 2 diabetes. The profound drop in C‐peptide (to 0.03 nmol/L) indicated near‐complete beta‐cell failure, consistent with ICI‐DM. Although diabetes‐associated autoantibodies were negative, the clinical and biochemical features were typical of an insulin‐deficient state, and he required insulin therapy—effectively transitioning from type 2 to insulin‐dependent diabetes. It should be noted that ICI‐DM does not necessarily require pre‐existing type 2 diabetes as a prerequisite; however, our case illustrates that patients with underlying type 2 diabetes may have reduced beta‐cell reserve and may therefore be particularly vulnerable to abrupt, immune‐mediated beta‐cell destruction induced by PD‐1 blockade. In this patient, the rapid onset of DKA after only three cycles of toripalimab—despite well‐controlled type 2 diabetes on oral hypoglycemic agents alone for one year—suggests that pre‐existing beta‐cell dysfunction, even if subclinical, may predispose to more rapid decompensation when immune tolerance is disrupted by checkpoint inhibition.

A meta‐analysis by Marchand et al. [5] showed that approximately 71% of ICI‐DM cases occur within 90 days of ICI initiation, with a median onset around the 4.5th treatment cycle. Our patient's DKA occurred slightly earlier (after three cycles), which may be attributable to his pre‐existing type 2 diabetes and reduced beta‐cell reserve. Notably, ICI‐DM can also develop months after ICI discontinuation [5], underscoring the need for prolonged glucose surveillance both during and after ICI therapy.

The concurrent development of hyperthyroidism in our patient suggests that PD‐1 inhibitors may induce immune‐mediated damage to multiple endocrine organs. A retrospective study by De Filette et al. [6] found that 65% of ICI‐DM patients carry the HLA‐DR4 genotype, a prevalence significantly higher than that in the general population. Furthermore, concomitant other endocrine irAEs (such as thyroid, adrenal, or pituitary abnormalities) are relatively common [7], indicating a broad propensity for autoimmune injury associated with these agents. In this case, hyperthyroidism developed approximately one month after initiation of toripalimab, manifesting with palpitations and elevated thyroid hormone levels, and the patient received methimazole therapy for one month before symptoms resolved. The sequential occurrence of thyroid autoimmunity followed by severe beta‐cell destruction within a short timeframe illustrates the broad‐spectrum autoimmune toxicity of PD‐1 inhibitors and supports the concept that damage to pancreatic beta‐cells and thyroid glands shares common immune‐mediated mechanisms.

This case has several clinical implications. First, clinicians should be aware that ICI‐DM can occur even in patients with pre‐existing type 2 diabetes, and DKA may be the first manifestation. Second, regular glucose monitoring should be maintained throughout ICI treatment and for a period after cessation. Third, the presence of one endocrine irAE (such as hyperthyroidism) should alert clinicians to the possibility of others, including ICI‐DM. Fourth, clinicians should be vigilant for early warning signs of ICI‐DM: any unexplained rise in plasma glucose levels after ICI initiation should prompt timely assessment of beta‐cell function and diabetes‐associated autoantibodies. A decline in C‐peptide levels or positivity for diabetes‐related autoantibodies should raise suspicion for impending ICI‐DM and increased risk of diabetic ketoacidosis, enabling earlier intervention before progression to ketoacidosis.

Several limitations of this case report should be acknowledged. First, as a single‐case report, the generalizability of our observations is limited, and larger cohort studies are needed to quantify the risk of ICI‐DM specifically in patients with pre‐existing type 2 diabetes. Second, the patient did not undergo regular glucose monitoring before admission, and only a single C‐peptide measurement was obtained after DKA onset, precluding an assessment of the trajectory of beta‐cell decline. Third, HLA genotyping was not performed, despite the known association between HLA‐DR4 and ICI‐DM, which could have further informed the underlying immunogenetic susceptibility. Finally, the follow‐up duration was relatively short, and long‐term glycemic outcomes and the persistence of insulin dependence remain to be determined.

## 6 Conclusion

This case demonstrates that ICI‐DM can occur abruptly in patients with pre‐existing type 2 diabetes, with DKA as the presenting manifestation. Early recognition and prompt insulin therapy are critical for favorable outcomes. Clinicians should maintain a high index of suspicion for ICI‐DM in patients receiving PD‐1/PD‐L1 inhibitors and should monitor glucose levels throughout therapy and after discontinuation. The concurrent development of hyperthyroidism in this case highlights the potential for ICIs to induce broad‐spectrum autoimmune endocrinopathies.

## Author Contributions

**Lingfen Zeng:** conceptualization, investigation, writing – original draft, writing – review and editing. **Jieming Sun:** data curation.

## Funding

The authors have nothing to report.

## Ethics Statement

Ethics approval was not required for this case report. The study was conducted in accordance with the Declaration of Helsinki.

## Consent

Written informed consent was obtained from the patient for publication of this case report and any accompanying images. A copy of the written consent is available for review by the Editor‐in‐Chief of this journal.

## Conflicts of Interest

The authors declare no conflicts of interest.

## Data Availability

Data sharing not applicable to this article as no datasets were generated or analysed during the current study.
